# Cam morphology, hip range of motion and hip pain in young skiers and soccer players

**DOI:** 10.1016/j.jsampl.2022.100005

**Published:** 2022-10-13

**Authors:** Anna Swärd Aminoff, Josefin Abrahamson, Carl Todd, Olof Thoreson, Cecilia Agnvall, Gauti Laxdal, Ricard Pruna, Pall Jonasson, Leif Swärd, Jón Karlsson, Adad Baranto

**Affiliations:** a Department of Orthopaedics, Institute of Clinical Sciences at Sahlgrenska Academy University of Gothenburg and Sahlgrenska University Hospital, Gothenburg, Sweden; b Sportmedicine Åre and Åre Ski High School, Sweden; c Orkuhúsið Orthopedic Clinic, Reykjavik, Iceland; d FC Barcelona Medical Services Excellent Center of Sports Medicine, Barcelona, Spain

**Keywords:** Femoroacetabular impingement, Sports medicine, Adolescent, Youth sports

## Abstract

**Objectives:**

To investigate hip pain, hip range of motion and the presence and size of cam morphology among young soccer players and skiers.

**Design:**

Cross-sectional study.

**Methods:**

The hip joints of young male soccer players and male and female skiers were examined clinically for hip range of motion (ROM) and with MRI (the presence of cam morphology). All participants answered questions about hip pain, debut age for training/competing and training frequency.

**Results:**

Clinical examinations were performed on 135 athletes (60 male soccer players, 40 male skiers and 35 female skiers), of which 93 athletes had additional MRIs. Mean age was 17.7 (SD 1.2) years. Cam morphology (α-angle ≥60°) was significantly less present in female skiers (3%) compared with male skiers (38%, p ​< ​0.001) and male soccer players (33%, p ​= ​0.03). The soccer players had significantly reduced internal and external hip rotation and self-reported hip pain, compared with the skiers. Female skiers had significantly greater hip ROM, compared with both male skiers and soccer players. A low positive correlation was found between cam morphology and hip pain in male skiers (r ​= ​0.42; p ​= ​0.014).

**Conclusions:**

Female skiers had a lower prevalence of cam morphology compared with the male groups while no difference was found between the male skiers and soccer players. Young male soccer players had significantly reduced internal and external hip rotation and a lower grade of self-reported hip pain compared with male and female skiers.

## 1 Introduction

The incidence of injuries in general and hip and groin injuries in particularly has reported to be 3.41/1000 and 0.4–1.1/1000 ​h of training and match play respectively, in elite soccer players [1,2]. Groin injuries stands for 12–16% of all injuries per season, making it one of the most common complaints in soccer [2,3]. In alpine skiing, injury incidence has shown to be 1.62–1.77/1000 skiing hours and 4.9–17.2/1000 skiing runs in young and adult elite skiers respectively [4,5]. The knee is the most common site of injury (41%), while the hip and groin accounted for 2% of the injuries [4]. Femoroacetabular impingement syndrome (FAIS) is a clinical disorder of the hip joint and is considered a major cause of hip pain in young athletes [6]. There are two types of morphologies associated with FAIS, i.e. cam morphology, presents as an aspherical shape of the femoral head at the femoral head-neck junction, and pincer morphology, present when the acetabular rim extends and creates local or global over-coverage of the femoral head [7]. However, for the diagnosis FAIS, imaging findings of cam and/or pincer morphology, clinical signs and symptoms are all need to be present [6]. This means that having imaging findings alone is not equal to the diagnosis of FAIS.

Clinical signs in patients with FAIS are reduced hip joint range of motion (ROM), particularly internal rotation in flexion, and positive hip impingement tests where the flexion, adduction, internal rotation test (FADIR) is the most commonly used test [6]. Symptoms related to FAIS are mainly motion- and/or position-related hip and/or groin pain, but also mechanical sensations of clicking, catching, locking and stiffness might be described by the patient [6]. In the moving hip (particularly flexion and IR), cam/pincer morphologies can cause an abnormal contact between the femoral head-neck junction and acetabulum. This mechanical abutment might secondarily lead to damages to the cartilage and labrum [8]. Cam morphology is also considered to be a major risk factor to hip joint osteoarthritis (OA) [9].

Radiological prevalence of cam morphology is higher among athletes (48–79%) compared with general population (5–55%) [10]. The prevalence of cam morphology in soccer players and alpine skiers have been reported to be 48–71% [[11], [12], [13], [14]] and 42–49% [15,16], respectively. Genetics and underlying pediatric conditions have been discussed as possible causes of cam morphology. However, mechanical factors, such as participation in high impacts sports during skeletal maturation, is today the most accepted theory [12,17,18].

The association between cam morphology and hip pain and symptoms is inconsistent and several studies have shown contradictory results [11,14,19,20]. This, in the combination with the fact that cam morphology is highly common in asymptomatic populations as well [3], making the pathway from having the morphology to develop clinical entity of FAIS (including hip and groin pain) complex. There are many questions still to be answered in terms of the etiology of cam morphology and why some individuals develop symptoms related to FAIS and others do not.

Therefore, it is of great interest to compare different groups of athletes, with different nationalities and sporting regimes, to further understand the etiology of cam morphology, clinical signs and symptoms.

The primary aim of this study was to compare hip ROM, hip pain and the presence and size of cam morphology between young Swedish male and female skiers and young soccer players from FC Barcelona U16 and the Icelandic U16 national team. The secondary aim was to investigate how cam morphology correlates with hip pain, debut age of training and competing and training frequency.

## 2 Methods

The sample group consisted of all young male soccer players (n ​= ​60, age range 15–18 years) from the Icelandic U16 national team and FC Barcelona U16 team, and elite male (n ​= ​40) and female (n ​= ​35) alpine and mogul skiers from the Åre Ski Academy (National Sports High school), Sweden (age range 15–21 years). Exclusion criteria were previously diagnosed hip, spine or pelvic injury, or previous surgery of the hips, spine or pelvis. One skier was excluded due to hip surgery prior to study start. Written consent was given by all individuals and by one or two parents if the participant was younger than 18 years of age. The present study was approved by the Regional Ethical Review Board, Gothenburg (ID number: 692-13).

The clinical examination included passive hip ROM for supine flexion, supine internal rotation (supine IR) and sitting internal (sitting IR) and external rotation (ER) and the FADIR test. All participants were examined by the same two examiners (co-authors ASA and CA) in a specific order to optimize the accuracy and standardization of the measurements. To assess hip ROM, a digital goniometer (HALO medical devices, Australia) [21] was used in combination with a universal goniometer with prolonged arms.

Supine hip flexion was assessed with the leg flexed in the sagittal plane to the point of initial resistance. Supine IR was examined with the hip and knee at 90° flexion in the sagittal plane, with the pelvis and opposite thigh stabilized, the hip was internally rotated to the point of initial resistance. Sitting IR and ER were measured with the participant in a sitting position, with a 1-cm thick pad underneath the most distal part of the thigh. The pelvis was controlled for a neutral position. The pelvis and opposite thigh were stabilized while the examined hip was first internally rotated to the point of initial resistance and then externally rotated. All measurements were repeated for the opposite hip. All hip ROM measures were recorded in degrees. The FADIR test was examined in the supine position with the test-leg passively brought into hip and knee flexion, internal rotation and adduction until resistance or pain/discomfort. A positive test was recorded if pain or discomfort was reported. Intra-observer reliability was tested with repeated measurements of 10 skiers with four months passed between the test occasions, showing a good to excellent level of agreement (intraclass correlation coefficient [ICC] 0.77–0.82). Inter-observer reliability was tested between ASA and CA in 10 skiers with a couple of hours in between, showing an ICC of 0.83–0.94 (good to excellent).

All skiers had their MRI examinations at Östersund Hospital, Sweden, and the soccer players were examined at the Icelandic Heart Association, Hjartavernd, Kópavogur, Iceland. The participants underwent MRI of both hips without contrast, using a standardized imaging protocol. Athletes who wanted information about their MRI, or had any divergent finding, were informed of their results. The MRI scanner used in Östersund, Sweden, was a GE Optima 450 Wide 1.5 ​T ​(GE Healthcare Bio-Sciences Corp, Piscataway, NJ, USA) and in Kópavogur, Iceland, a Signa Twin-speed; EXCITE 16 channel system 1.5 ​T ​(GE Healthcare Bio-Sciences Corp, Piscataway, NJ, USA) was used. Cor T2 Fat Sat and Ax 3D Cube sequences were obtained angled to the femoral neck using a coil surface of HD 8 Channel Cardiac Array (GE Healthcare Bio-Sciences Corp). The α-angle and the status of the growth plate were evaluated and measured by two radiologists, one specifically measured the soccer players, whilst the other performed measurements of the skiers. Both were under the guidance of the same senior consultant radiologist. The status of the growth plate was evaluated as being either closed or open, based on the appearance of the capital femoral growth plate on MRI using the same method as Siebenrock et al. [17]. The α-angle is the most common measure to quantify the presence and size of cam morphology, and was measured according to Nötzli et al. [22]. This measurement was performed at seven locations around the femoral head, from 9 o'clock (posterior) to 3 o'clock (anterior, 180°). To define the presence of cam morphology, cut-off values of the α-angle has been varied between 50.5 and 83° [10]. By using a higher cut-off value, it might optimize its discriminative power and increase specificity, but with a higher cut-off value, the sensitivity decreases. However, in the Lisbon Agreement on Femoroacetabular Impingement Imaging from 2020, a cut-off value of 60° was recommended, as higher values have been reported be clinical more relevant [23]. Therefore, a cam morphology was considered present when the α-angle was 60° or higher. Inter-observer reliability was tested between the two radiologists of 15 randomly selected skiers and soccer players MRI scans. An ICC of 0.46–0.81 (poor to good) was shown. For intra-observer reliability, repeated measurements of 15 randomly selected MRI scans, with four weeks between, was performed by the two radiologists separately, showing a good to excellent agreement (ICC 0.83–0.97).

The athletes had to answer questions about hip pain, training frequency (*Number of training days/week?* And *Number of training hours/week?*), debut age of training (*At what age did you start to train skiing/soccer?*) and competing (*At what age did you start to compete in skiing/play soccer matches?)*. Hip pain was considered present if the athlete had positive answer/-s at any of the three following questions: *Do you have, or have had, hip pain?* (“Yes, at present”), *Do you have, or have had, hip pain the last* 12 months*?* (Yes) or *Do you have, or have had, hip pain the last 7 days?* (Yes). Hip pain was defined as pain in the groin or hip in either the right or left hip.

IBM SPSS Statistics, version 26 (IBM Corp) was used in the analysis of the data. Data was expressed in terms of mean and standard deviation (SD) or as number and percentage (%) unless specified. Due to skewed distribution for debut age of training and competing these variables was expressed in terms of median and interquartile range (IQR). One-way ANOVA with Bonferroni post hoc test was used for comparisons of demography, hip ROM and the α-angle between groups. Chi [2] test was used for group comparisons of the categorical variables; prevalence of cam morphology (α-angle ≥60° at any clock position), FADIR test and hip pain. Spearman rank correlation coefficient was used for correlation analysis between hip pain vs. the presence of cam morphology (α-angle ≥60° at any position) and size of cam morphology. Values ​< ​0.3 indicate negligible correlation; between 0.3 and 0.5 low correlation; 0.5–0.7 moderate correlation; 0.7-0-9 high correlation and 0.9–1.0 very high correlation [24]. All tests were two-sided and p-values of <0.05 were considered significant. Mann–Whitney U test were used to compare debut age of training and competing with the presence of cam morphology. A 2-way random effect with single measurement and absolute agreement model with 95% confidence interval was used.

## 3 Results

A total of 135 athletes, 100 males and 35 females, with a mean age of 17.7 (SD 1.2) years were included. Table 1 shows the characteristics of all athletes and comparisons between groups. All skiers except one were Swedish born, while the soccer players were from all over the world. The majority of all athletes had nine or more training hours/week, distributed over 5–6 days. Median age of the debut of training and debut age of competition ranged between 5-6 years and 7–8 years, respectively.

**Table 1:** Participant characteristics for all athletes and between groups [1].

	All athletes	Female skiers	Male skiers	Male soccer players	Sign
**No. of participants**	135	35	40	60	–
**Country of birth, number**					
Sweden	74	35	39	0	
Iceland	30	0	0	30	
Various countries	31	0	1	30	
**Age, years**^a^	17.7 (1.2)	18.3 (1.0)	18.2 (1.2)	17.1 (0.9)	d^,^^f^
**Height, cm**^a^	177 (8.0)	169 (5.8)	179 (6.8)	180 (6.6)	d^,^^e^
**Weight, kg**^a^	70.4 (8.4)	65.0 (7.1)	74.0 (8.7)	71.1 (7.6)	d^,^^e^
**BMI kg/m2**^a^	22.5 (1.9)	22.8 (2.2)	23.1 (2.0)	22.0 (1.3)	f
**Training days/week**^a^	5.6 (0.7)	5.9 (0.6)	5.7 (0.6)	5.4 (0.8)	e
**Training hours/week**^a^^**,**^^b^					e
3–5 ​h	1 (0.8)	1 (2.9)	0	0	
6–8 ​h	27 (20.1)	12 (34.3)	6 (15.4)	9 (15.0)	
9–11 ​h	42 (31.3)	12 (34.3)	16 (41.0)	14 (23.3)	
>11 ​h	64 (47.8)	10 (28.5)	17 (43.6)	37 (61.7)	
**Debut age of training, years**^c^	6 (5–7)	6 (5–8)	6 (5–8)	5 (4–6)	e^,^^f^
**Debut age of competing, years**^c^	7 (6–9)	7 (6–10)	8 (7–11)	7 (6–8)	e^,^^f^

Values in mean (SD) unless specified.

a One-way ANOVA with Bonferroni post hoc test for comparison between groups.

b Values in numbers (%).

c Mann Whitney U test, values in median (IQR).

d Indicates significant difference between female skiers and male skiers.

e Indicates significant difference between female skiers and soccer players.

f Indicates significant difference between male skiers and soccer players, at p-value <0.05.

**Fig. 1:**
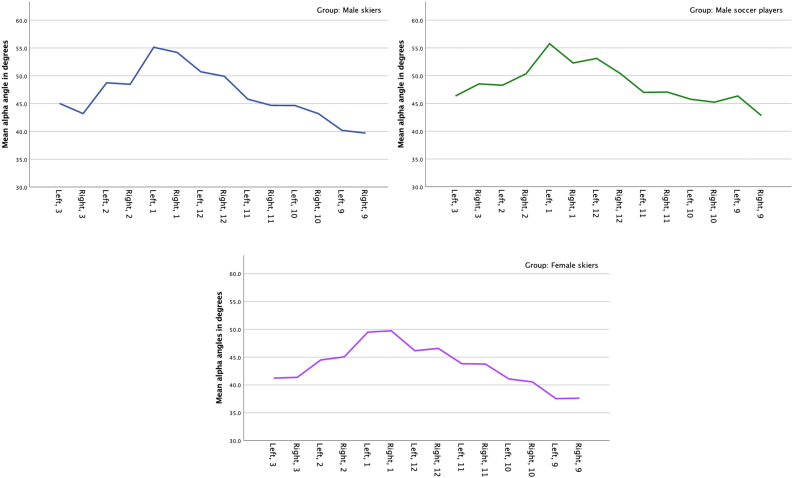
Line graphs of the mean α-angle in the left (Left) and right (Right) hip at the 3 to 9 o'clock positions in male skiers (top left), male soccer players (top right) and female skiers (bottom).

One-hundred and seventy-two hips in 93 athletes were analyzed using MRI, for the presence of cam morphology. Thirty soccer players and twelve skiers (six males and six females) were not able to participate, or did not show up for the MRI examination, and remaining missing MRI data (14 hips) were due to bad imaging quality that precluded interpretation. All participants investigated with MRI had closed growth plates. Table 2 shows the distribution of cam morphology between the groups. There was no statistically significant difference in cam morphology prevalence between male skiers (38.2%) and the soccer players (33.3%), while female skiers had a significantly lower prevalence (3.4%) compared with both the male skiers (p ​< ​0.001) and soccer players (p ​= ​0.03). The α-angle had the highest values at the 1 o'clock position in all groups (Fig. 1). The α-angle did not differ between the male groups of athletes except for the left hip at 9 o'clock-position (p ​= ​0.003). Female skiers had a significantly lower α-angle compared with male skiers in both the right and left hip at 12 (p ​= ​0.03 and p ​< ​0.001), 1 (p ​= ​0.004 for both hips) and 2 (p ​= ​0.04 and p ​= ​0.003) o'clock, and at 3 (p ​= ​0.02) o'clock in the left hip. Compared with male soccer players, female skiers had a significantly lower α-angle at all clock-positions (p-value ranged between <0.001 and 0.046) except for 11 o'clock (both right and left) and 1 o'clock (right hip only) (Fig. 1).

**Table 2:** Comparisons of cam morphology (60°) prevalence at any position.

	Female skiers	Male skiers	Male soccer players	Sign
Right hip	0 (0)	7 (20.6)	4 (20.0)	a^,^^b^
Left hip	1 (3.4)	10 (29.4)	8 (29.6)	a^,^^b^
Either hip (right or left)	1 (3.4)	13 (38.2)	10 (33.3)	a^,^^b^
Bilateral (right and left)	0 (0)	4 (11.8)	2 (6.7)	n.s.

Values in number (%) unless specified. ^1^Chi^2^ test for comparison between groups.

a Indicates significant difference between male and female skiers.

b Indicates significant difference between soccer players and female skiers at p-value <0.05.

Table 3 demonstrates hip joint ROM and the FADIR test between the groups. The soccer players had significantly reduced IR (both in the supine and sitting position) and ER compared with male skiers (p ​< ​0.001), while there was no difference for hip flexion. Female skiers had a significantly greater hip ROM in all directions compared with all other groups, except for the right ER compared with male skiers. No differences were found for the FADIR test between the groups.

**Table 3:** Hip ROM in degrees (°) and FADIR test between groups.^a^

		Female skiers (n ​= ​34)	Male skiers (n ​= ​37)	Male soccer players (n ​= ​60)	Sign
Flexion	Right	121.4 (10.5)	115.1 (7.5)	114.4 (6.7)	c^,^^d^
	Left	123.3 (7.9)	115.0 (8.2)	115.5 (6.4)	c^,^^d^
Supine IR	Right	32.8 (8.7)	24.7 (7.9)	10.9 (5.5)	c^,^^d^^,^^e^
	Left	35.5 (8.7)	24.9 (7.6)	14.7 (7.7)	c^,^^d^^,^^e^
Sitting IR	Right	40.7 (7.3)	27.7 (8.0)	22.2 (7.9)	c^,^^d^^,^^e^
	Left	42.4 (7.8)	29.5 (7.3)	21.6 (9.7)	c^,^^d^^,^^e^
ER	Right	38.5 (8.4)	35.4 (6.5)	25.9 (5.5)	d^,^^e^
	Left	38.6 (6.4)	34.4 (5.5)	26.8 (6.3)	c^,^^d^^,^^e^
FADIR^b^	Right	27 (79.4)	30 (81.1)	46 (76.7)	n.s.
	Left	22 (64.7)	30 (81.1)	38 (63.3)	n.s.

Values in mean (SD).

a One-way ANOVA with Bonferroni post hoc test for comparison of ROM between groups and Chi^2^ test for FADIR. Supine IR, internal rotation in supine position; Sitting IR, internal rotation in sitting position; ER, external rotation in sitting position.

b Number (%) with pain/discomfort.

c Indicates significant difference between female skiers and male skiers.

d Indicates significant difference between female skiers and soccer players.

e Indicates significant difference between male skiers and soccer players, at p-value <0.05.

Sixteen male skiers (41%), seven soccer players (12%) and 18 female skiers (51%) reported hip pain. This differed between soccer players and skiers (p ​< ​0.001), but not between female and male skiers.

There was a low, positive correlation between hip pain and cam morphology (r ​= ​0.42; p ​= ​0.014) in male skiers. No correlations were found between hip pain and cam morphology in female skiers, in male soccer players or in the analyze of all athletes together. No correlation was found between the size of the α-angle and hip pain in the analyze of all athletes together or when stratified by group. Debut age of training and competing or training days/week and training hours/week did not differ in those athletes with or without cam morphology.

## 4 Discussion

The primary aim of this study was to compare hip ROM, hip pain and the presence and size of cam morphology between young male and female skiers and young soccer players. The prevalence of cam morphology did not differ between male skiers (38%) and soccer players (33%), while female skiers had significantly lesser amount of cam morphology (3%).

Sports with high intensity that impose a greater degree of loading on the hip joint, particularly during the pubertal growth spurt, are considered a possible cause for the development of cam morphology [12,25]. Despite the fact that skiing and soccer are two different sports, with different demands and loadings on the hips, this does not seem to differentiate male skiers and soccer players in the development of cam morphology in this study. However, the prevalence of cam morphology is lower in this study compared with previous studies of young soccer players (range 48–60%) [11,12], while the results for male skiers are comparable to a previous study (42%) [16]. Agricola et al. [12] and van Klij et al. [11] investigated 63 and 49 young academic male soccer players with mean age of 13 and 15 years, respectively. Although both studies used an α-angle of ≥60° to define the presence of cam morphology, as in this study, they used AP pelvic and frog-leg lateral radiographs (and this study used MRIs) which may be a part of the difference in results. In the study by Philippon et al. [16], young ice-hockey players and skiers (10–18 years) were examined with MRIs for the presence of cam morphology with a cut-off value for the α-angle of ≥55°. This lower cut-off value, as compared with ≥60° used in this study, is probably the reason why they had a slightly higher prevalence compared with this study. It is tempting to suggest that the development of cam morphology may in part be related to the time frame when the hip joints are most vulnerable to high level loading, rather than the different variables of load that the hip joints may be subjected to in soccer and skiing.

The fact that female skiers had a lower prevalence of cam morphology, and lower α-angles at almost every clock-position, compared with both the male groups correlates well with previous studies [13,15,19]. These studies have shown a higher prevalence of cam morphology among male compared with females. Females enter the pubertal growth spurt earlier than males, and have an earlier closure of their growth plate of the proximal femur [26]. Therefore, it is tempting to speculate that when the demands of training and competing increases the female growth plate is already closed and therefore no cam morphology develops. Despite the fact that females had lower α-angles, the α-angle had the highest values at the 1 o'clock position in all groups (Fig. 1). This result is in line with previous studies that have shown that a cam morphology mainly develops at the antero-superior part (1–2 o'clock) of the femoral head [23].

The soccer players had significantly reduced hip IR and ER compared with both male and female skiers, while female skiers had significantly greater hip ROM in almost all ranges compared with all three groups of males (Table 3). Females are known to have a generally greater level of joint laxity, which correlates well with the findings in this study [27]. Another explanation might be the lower prevalence, and size of, cam morphology present in females in this study.

The results of hip IR in both skiers and soccer players are comparable to previous studies of athletes (11–35°) [28,29]. Moreover, the result of hip ER in the skiers in this study was also in accordance with previous studies of athletes (30–50°) [28,29]. However, why the soccer players had less hip ER compared with previous studies might be explained by different methods used. This study used a sitting position for ER measurements, while the above-mentioned studies used a supine position. The same two examiners did all the tests according to the same testing procedure in all the included groups. The differences in hip IR and ER between the male skiers and soccer players, in this study, are therefore possibility related to the different biomechanical demands that these two sports impose rather than the methodological differences. Moreover, hip pain was not recorded in conjunction with the hip ROM examinations, which might also be an explanation to the differences observed between skiers and soccer players in this study.

The secondary aim was to investigate how cam morphology correlates with hip pain, debut age of training and competing and training frequency. Only in male skiers a positive, low correlation (r ​= ​0.42; p ​= ​0.014) was found between hip pain and cam morphology (defined as an α-angle ≥60°). No other correlations were found between either the presence or size of cam morphology. These inconclusive results are comparable to previous studies where the association between cam morphology and hip pain and symptoms have been questioned, with conflicting evidence being reported [11,14,19,20]. In the study by Gosvig et al. [19], no association was found between cam morphology (defined as an α-angle ≥83° in males and ≥57° in females) and self-reported pain in 3202 participants of a general population. Mosler et al. [14] also found no association, in their longitudinal study between cam morphology (defined as an α-angle ≥60°) or large cam morphology (α-angle ≥78°) and groin injuries in elite soccer players. In the study by Larson et al. [20] a larger cam morphology, defined as having a higher α-angle, with no cut-off value, was associated with hip and groin pain. However, in the study van Klij et al. [11] only a large visual cam morphology, defined as having a visually prominence at the femoral head-neck junction, was associated with hip and groin pain, but not with symptoms (based on HAGOS). In contrast, a large cam morphology defined as an α-angle ≥78° was not associated with either hip and groin pain or symptoms, in the same study [11].

A larger cam morphology might be more likely to cause a rapid intra-articular injury and symptoms rather than a smaller one. However, based on the contradictory results from this study and previous studies, it is still unclear how cam morphology may or may not be associated with hip and groin pain. One explanation, to the non-association in this study, might be related to the fairly low mean α-angles shown in this study (Fig. 1) or the fact that the participants were relatively young. Cam morphology is developed during growth spurt, approx. between 12 and 14 years of age and continue to develop until the growth plate is closed [12,17,25]. The mean age of participants in this study was between 17.1 and 18.3 years, and the duration of cam morphology might have been too short to create pain and/or symptoms. The pathway from having cam morphology to develop clinical entity of FAIS (including symptoms and clinical signs) is not straight forward, and include other hip and pelvic variations (e.g. femoral version, acetabular morphology, spino–pelvic parameters), soft-tissues, the activity the athlete perform and other person-specific factors [23].

Another explanation might be the low number of soccer players reporting hip pain (12%), compared with both male and female skiers (41% and 51% respectively). This is somewhat surprising result considering that hip and groin pain has been shown to be one of the most common complaints in soccer players (prevalence of 48% per season) [3]. In contrast, hip and groin injuries seems to be fairly low in alpine skiers, where these injuries accounted for only 2% of all injuries in a cohort of young elite alpine skiers during 3–4 years [4]. However, there is a lack of studies investigating overuse injuries in alpine skiers and, therefore, hip and groin injuries with insidious onset of pain and symptoms might be overlooked and thus under-reported. One explanation why the soccer players reported less hip pain, may be that previously injured soccer players may have already been dismissed from the team, or they may have under-reported hip pain in fear of losing their position in the team. This contrasts with the skiers that came from a high school and were not subjected to any such selections or risk of dismissal. Another explanation could be that the soccer players were slightly younger, giving them one year extra to develop hip pain.

A limitation of this study is the lack of MRI examinations from the soccer players. Perhaps a greater number of MRI's from the soccer players, may have helped develop a better understanding of the underlying cause for the reduced hip IR and ER that they demonstrated. Physical examination is also dependent on the examiner however, in the present study, to limit variations in results, the same two examiners performed all measurements. This may also be seen as a strength of this study, that the same two examiners clinically examined all athletes according to the same standardized protocol. Another strength was that all athletes were shown to have closed femoral growth plates, making them all comparable with each other [25]. Although good to excellent intra-rater reliability was shown for the radiologists, the levels of inter-rater reliability between the two radiologists might affect the outcomes in the interpretation of cam morphology between the groups of soccer players vs. skiers.

## 5 Conclusion

Young male soccer players had significantly reduced hip internal and external rotation, compared with male and female skiers. Male skiers and soccer players had a higher prevalence of cam morphology compared with female skiers, while both female and male skiers did report hip and groin pain in higher degrees compared with soccer players.

## 6 Practical implication

• Male soccer players were shown to have reduced hip internal and external rotation, which might increase the risk of injuries [30].
• Female skiers had a lower prevalence of cam morphology, smaller α-angles and greater hip ROM, but did report a higher degrees of hip pain.
• The prevalence of cam morphology was high in male athletes in both soccer and skiing.
• Skiers (both male and female) reported hip pain to a greater proportion compared with soccer players.

## Funding information

The financial support of The Medical Society of Gothenburg, Sweden, Carl Bennet AB and, grants from the Swedish state under the agreement between the Swedish government and the county councils, the ALF agreement (Adad Baranto, ID number 238801 and Jon Karlsson, ID number 74020) had no involvement in the study design, data collection, analysis or submit decisions. This research did not receive any specific grant from funding agencies in the public, commercial, or not-for-profit sectors.

## Confirmation of ethical compliance

The authors confirm that the study has been performed according to, and followed, the Ethical Guidelines. Written consent was given by all athletes and by one or two parents if the participant was younger than 18 years of age. The study was approved by the Regional Ethical Review Board, Gothenburg (ID number: 692-13).

## Declaration of competing interest

The authors declare that they have no known competing financial interests or personal relationships that could have appeared to influence the work reported in this paper.
